# Serum Metabolomics and Proteomics to Study the Antihypertensive Effect of Protein Extracts from *Tenebrio molitor*

**DOI:** 10.3390/nu14163288

**Published:** 2022-08-11

**Authors:** Roberto Stella, Caterina Peggion, Caterina Bergantin, Giancarlo Biancotto, Maria Frosini, Elena Dreassi, Paola Marcolongo, Anna Maria Aloisi, Federica Pessina

**Affiliations:** 1 Istituto Zooprofilattico Sperimentale delle Venezie, Department of Chemistry, viale dell’Università, 35020 Padova, Italy; 2 Department of Biomedical Sciences, University of Padova, via Ugo Bassi 58/b, 35131 Padova, Italy; 3 Department of Life Sciences, University of Siena, via A. Moro, 53100 Siena, Italy; 4 Department of Biotechnology, Chemistry and Pharmacy, University of Siena, via A. Moro, 53100 Siena, Italy; 5 Department of Molecular and Developmental Medicine, University of Siena, via A. Moro, 53100 Siena, Italy; 6 Department of Medicine, Surgery and Neuroscience, University of Siena, via A. Moro, 53100 Siena, Italy

**Keywords:** functional food, multi-omics, blood pressure, SHR rats, insect protein supplementation

## Abstract

Hypertension is the leading risk factor for premature death worldwide and significantly contributes to the development of all major cardiovascular disease events. The management of high blood pressure includes lifestyle changes and treatment with antihypertensive drugs. Recently, it was demonstrated that a diet supplemented with *Tenebrio molitor* (TM) extracts is useful in the management of numerous pathologies, including hypertension. This study is aimed at unveiling the underlying mechanism and the molecular targets of intervention of TM dietary supplementation in hypertension treatment by means of proteomics and metabolomics techniques based on liquid chromatography coupled with high-resolution mass spectrometry. We demonstrate that serum proteome and metabolome of spontaneously hypertensive rats are severely altered with respect to their normotensive counterparts. Additionally, our results reveal that a diet enriched with TM extracts restores the expression of 15 metabolites and 17 proteins mainly involved in biological pathways associated with blood pressure maintenance, such as the renin–angiotensin and kallikrein–kinin systems, serin protease inhibitors, reactive oxygen scavenging, and lipid peroxidation. This study provides novel insights into the molecular pathways that may underlie the beneficial effects of TM, thus corroborating that TM could be proposed as a helpful functional food supplement in the treatment of hypertension.

## 1. Introduction

About a third of adults are affected by idiopathic hypertension, called essential hypertension, one of the most common contributors to mortality in the developed and developing world [1,2]. Hypertension represents an important issue in public health because of its increasing prevalence and its role in the development of several other diseases [3,4,5]. In fact, increased blood pressure is one of the most important comorbidities for the development of cardiovascular and cerebrovascular diseases, renal and heart failure, and stroke [6,7]. The mechanism of hypertension is complex as it arises from multiple genetic and environmental factors. Indeed, except for a few cases of patients with either renal or adrenal disease, there is no clear single identifiable cause in 95–97% of hypertension cases. Hypertension is probably caused by impairment of one of the numerous mechanisms involved in the maintenance of normal blood pressure, which can differ among individuals. Such mechanisms may include, for example, (i) the renin–angiotensin–aldosterone system, consisting in a cascade in which angiotensinogen is converted by angiotensin converting enzyme (ACE) into angiotensin II, a potent vasoconstrictor that causes the rise of blood pressure; (ii) stimulation of the sympathetic nervous system, causing arteriolar constriction; (iii) endothelial dysfunction; and (iv) the production of vasoactive substances that affect sodium transport and vascular tone thus regulating blood pressure [8].

The control of blood pressure is essential to reduce the risk of associated diseases. While lifestyle interventions, such as dietary modification and weight loss, have proven to be useful to prevent hypertension, antihypertensive drugs are extensively used in its treatment (i.e., calcium channel- and beta-blockers, thiazide-type diuretics, angiotensin receptors blockers, as well as ACE- or renin-inhibitors) [9,10,11,12]. In particular, captopril is a medication used worldwide in the management of hypertension [13]. It is a specific competitive inhibitor of ACE, and the lowered production in angiotensin II causes a reduction in blood pressure and cardiovascular remodeling both in humans with essential hypertension and in different animal models of arterial hypertension [14]. However, although there are effective and safe antihypertensive drugs, hypertension persists uncontrolled in most patients. For this reason, the development of new antihypertensive molecules to be used as a functional ingredient in fortified food and/or as a dietary supplement to lower blood pressure can be an interesting and useful approach to reduce the related cardiovascular risk.

Much of the current knowledge on mechanisms of essential hypertension has been provided by studies performed in the most exploited animal model of hypertension: spontaneously hypertensive rats (SHR) and the normotensive control strain, Wistar–Kyoto rats (WKR). Indeed, the SHR animal model is one of the closest to human essential hypertension in terms of pathogenesis, peripheral vascular resistance changes, and cardiovascular complications, and it is also useful in studies for the development of new antihypertensive drugs [15,16].

In this context, SHRs have been used as a model of hypertension to examine the antihypertensive effects of several herbal extracts such as Tengfu Jiangya (a combination of *Uncaria rhynchophylla* and raphani semen), Alismatis Rhizome (Oriental water plantain rhizome), and aged garlic extracts, in order to discover therapeutic targets of hypertension by applying metabolomics and proteomics techniques [17,18,19,20]. Such omics approaches represent a powerful and high throughput technique already applied in medical diagnostics and basic research [21,22]. Both techniques have been used to explore the pathophysiology of diseases [23], to provide insight into endogenous modifications in response to pharmacological treatments [24,25], and to discover biomarkers in biological systems [26].

Recently, we found that supplementation of the SHR diet with protein extracts from defatted larvae of *Tenebrio molitor* (TM) prevented spontaneous rise in blood pressure, reduced heart rate and coronary perfusion pressure, and exerted neuroprotection, thus demonstrating the possibility to restore physiological status after nutritional interventions [27].

The inclusion of edible insects, such as TM larvae, in the human diet has been shown not only to improve the nutritional quality of food due to their high micro- and macronutrient levels (rich in essential amino acids, polyunsaturated fatty acids, and a variety of vitamins and minerals, such as calcium, zinc, iron, magnesium, riboflavin, pantothenic acid, and biotin) but also to provide meaningful pharmacological properties against several pathologies, including hypertension [27,28,29,30].

Insects are already consumed in many countries in Asia, Africa, and South America. Nonetheless, their consumption elsewhere is limited due to a skeptical attitude towards this kind of food, possible chemical and microbiological risks, or in some cases, lack of specific legislation [28].

In the present study, we performed an in-depth analysis of the serum proteome and metabolome of SHRs whose diet was supplemented with protein extracts from TM in comparison with their normotensive WKY counterparts to explore the physiological adaptation occurring at the protein and metabolite level and to evaluate potential protective effects of dietary supplementation with TM. These effects on serum metabolome and proteome profiles were compared to those elicited by the pharmacological treatment with captopril.

Our results demonstrated that serum from SHRs presents alterations of the metabolic fingerprint and of proteins principally involved in the regulation of peptidase activity and oxidative stress. Interestingly, dietary supplementation with TM extract reversed some of these changes, in a similar manner to the pharmacological treatment with captopril, thus suggesting that a dietary approach based on the introduction of edible insects may be useful in the prevention/treatment of hypertension.

## 2. Materials and Methods

### 2.1. Chemicals and Materials

Formic acid (98%) and LC-MS grade acetonitrile and water were purchased from Fisher Scientific (Fair Lawn, NJ, USA). Acetone, trichloroacetic acid (TCA), and glacial acetic acid were obtained from Carlo Erba (Milan, Italy). LC-MS grade methanol and reference analytical standards used for the identification of docosanoic acid were purchased from Sigma Aldrich (St. Louis, MO, USA). Isotope-labeled internal standards (creatine-D_3_, leucine-5,5,5-D_3_, l-tryptophan-2,3,3-D_3_, indole-2,4,5,6,7,3-acetic acid-D_5_, 1,14-tetradecanedioic acid-D_24_) were from Merck (St. Louis, MO, USA) or from CDN Isotopes (Québec, Canada). Micro Lowry protein assay kit, urea, tri-hydroxy-methyl-aminomethane (Tris), iodoacetamide (IAA), and dithiothreitol (DTT) were purchased from Sigma Aldrich (St Louis, MO, USA). Sequencing-grade modified trypsin from porcine pancreas was purchased from Promega (Madison, WI, USA). BioPure C_18_ spin columns were obtained from The Nest Group Inc. (Southborough, MA, USA).

### 2.2. TM Preparation

Defatted larvae used in this study were obtained by extraction in ethanol as already described in [27]. Fat content was determined by the Soxhlet extraction method, while the Kjeldahl method was used to determine crude protein. Proteins extracted as water-soluble fraction (supernatant) or as water-insoluble fraction (pellet) were characterized by SDS-PAGE and LC-MS/MS. The nutritional value and other details of defatted TM are reported in [27]. Mucedola Srl (Italy) prepared the standard diet (4RF25, for composition see www.mucedola.it) supplemented with larva protein or with captopril as previously reported [27]. All toxicological/genotoxicological aspects were previously assessed as described in [27].

### 2.3. Animal Experiment

Nine-week-old male spontaneously hypertensive rats (SHRs, *n* = 12) and age-matched Wistar–Kyoto (WKY) rats (*n* = 12) as the normal blood pressure control group of SHRs were purchased from Charles River (Lecco, Italy). Two to four rats were housed in each cage in a room maintained at constant temperature (23–24 °C) and humidity (50–60%), on a 12 h light/dark cycle, with free access to food and water.

At ten weeks of age, when hypertension develops, hypertensive SHR and normotensive WKY rats were randomly divided into three groups of four rats and fed different diets. The control group was fed with a standard diet (SD) for four weeks. The remaining groups were fed with a standard diet supplemented with 4.5% of larva protein from TM or supplemented with 100 mg/kg of captopril (C). On the basis of the average daily food intake and rat body weight, a daily dose of ~ 2.9 g/kg of TM and ~ 8 mg/kg body weight of captopril was estimated [27]. The captopril dose assumed by animals was reported to reduce blood pressure values [31,32] and to inhibit plasma ACE activity [33] in SHR, and it is administered to treat mild-to-moderate hypertension in patients [34]. Systolic blood pressure was measured before treatment (time 0, basal value) and once a week by the non-invasive “tail-cuff” method, recorded with a digital PowerLab data acquisition system (PowerLab 8/30, ADInstruments, Castle Hill, Australia) and analyzed using LabChart 7.3.7 Pro (Power Lab, ADInstruments) [35]. Three consecutive blood pressure readings were taken between 9 am and 1 pm. At the end of this four-week period, all rats were anesthetized and blood samples were collected.

All aspects of animal care and experimentation were carried out in compliance with European and Italian (D.L. 26/2014) laws concerning the care and use of laboratory animals. The authors’ institution has been accredited for the use of experimental rats by the Italian Ministry of Health (157/2017-PR).

### 2.4. Sample Preparation

Blood (~0.5 mL) was collected in tubes not containing anti-coagulant agents and was allowed to clot. Samples were centrifuged at 3000× *g* for 10 min at 4 °C and serum samples were aliquoted and stored at −80 °C pending analysis.

### 2.5. LC-HRMS Metabolomics Analysis

One aliquot of serum samples was taken from each of the 24 samples under investigation, and they were thawed in melting ice before analysis. Proteins were precipitated in a plastic tube by adding 150 µL of methanol to an aliquot of 50 µL of serum. Each sample was vortexed for 1 min and kept at −80 °C for 2 h, and then the samples were centrifuged at 21,000× *g* for 10 min at 4 °C. After centrifugation, 75 µL of supernatant were transferred to a glass vial, and 25 µL of labeled internal standards dissolved in water at a concentration of 4 ng µL^−1^ were added prior to liquid chromatography coupled with high resolution mass spectrometry (LC-HRMS) analysis.

Samples were analyzed in LC-HRMS using an Ultimate 3000 UHPLC system (Thermo Scientific, Waltham, MA, USA) coupled with a quadrupole-orbitrap (Q-Exactive) high-resolution mass spectrometer (Thermo Scientific, USA). Metabolomics profiling was carried out using reversed phase liquid chromatography (RPLC) and electrospray ionization both in positive and negative modes. Chromatographic separation of metabolites was performed on a Hypersil Gold C_18_ column (100 × 2.1 mm, 1.9 µm, Thermo Scientific, USA) kept at 35 °C. The mobile phases were LC-MS grade water (A) and LC-MS grade acetonitrile (B), both containing 0.1% acetic acid (*v*/*v*), the flow rate was 0.4 mL min^−1^, and the injection volume was 5 µL. The chromatographic gradient was: 0–2.5 min, 5% B; 2.5–15.5 min, 5–100% B; 15.5–20 min, 100% B; 20–20.5 min, 100–5% B; 20.5–25 min, 5% B.

Data were acquired in full scan mode at a resolving power of 70,000 full width at half maximum (FWHM) at 200 *m*/*z*. The mass range of mass spectra was between 70 and 1000 *m*/*z*, the automatic gain control target was set to 3 × 10^6^ ions, and the inject time was set to 200 ms. Sheath gas flow rate was 40 arbitrary units and auxiliary gas flow rate was 10 arbitrary units. Capillary temperature and heater temperature were 325 °C. The capillary voltage was 3.5 kV in positive polarity electrospray and 3.0 kV in negative polarity electrospray, and the S-lens value was 50 V for both positive and negative ionization mode.

Each analytical session was carried out by randomizing the injection order of serum samples deriving from different groups and using QC samples that were injected during the analytical batch to normalize instrumental response [36]. We used isotopically labeled internal standards to check the LC-HRMS system performance by verifying the consistency of chromatographic retention time and mass deviation in each sample injected along the analytical session. Metabolites in serum samples were analyzed by means of LC–HRMS using both positive and negative ionization polarity. Chromatographic peaks alignment and integration was performed using Compound Discoverer 2.1 software. Normalized area values, retention time, and *m*/*z* values of detected metabolites were exported into an Excel spreadsheet for further analysis.

### 2.6. Proteomics Analysis: Protein Precipitation and Trypsin Digestion

Serum samples were thawed on ice-cold water, and then the total protein content was calculated with the Lowry assay. Removal of albumin was achieved by using TCA/acetone precipitation [37]. An aliquot containing 500 µg of total proteins was diluted by adding a suitable amount of water to a final volume of 50 µL. Subsequently, 8 volumes (i.e., 400 µL) of ice-cold TCA/acetone (10%, *v*/*v*) were added to the diluted serum samples for albumin removal. Samples were vortexed and incubated at −20 °C overnight. Samples were then centrifuged at 16,000× *g* for 5 min at 4 °C to achieve precipitation of highly abundant proteins and the supernatant was discarded. The protein pellet was washed by adding 1 mL of ice-cold acetone, and then samples were vortexed and centrifuged again at 16,000× *g* for 5 min at 4 °C.

The supernatant was discarded, and the pellet was dissolved in 60 µL of 8 M urea containing 100 mM Tris-HCl pH 8. Reduction of disulfide bonds was carried out by adding 20 µL of 100 mM DTT and incubating samples for 1 h at room temperature (DTT final concentration equal to 25 mM). Alkylation of reduced cysteines was achieved by adding 20 µL of 275 mM IAA and incubating samples for 45 min at room temperature in the dark (IAA final concentration equal to 55 mM). Then, 375 µL of 100 mM Tris-HCl pH 8 were added to each sample, and protein digestion was performed by adding 2.5 μg of sequencing grade modified trypsin and incubating samples at 37 °C overnight.

After protein digestion, samples were acidified by adding formic acid to a final concentration of 1% to stop the reaction. Peptides were loaded on a BioPure C_18_ spin column (The Nest Group Inc., USA) to eliminate salts. After activation with 400 µL of acetonitrile, columns were equilibrated twice with 400 µL of water containing 0.1% formic acid (*v*/*v*) and then peptides were loaded. After two washing steps with water containing 0.1% formic acid (*v*/*v*), digested peptides were eluted twice with 100 μL of 75% ACN in water (*v*/*v*) containing 0.1% formic acid (*v*/*v*). Purified digested peptides were dried, suspended with 50 μL of 5% ACN in water (*v*/*v*) containing 0.1% formic acid (*v*/*v*), and transferred to an autosampler vial for the LC-HRMS/MS analysis.

### 2.7. LC-HRMS/MS Proteomics Analysis and Database Search

Serum digests were analyzed by means of an untargeted proteomics approach based on LC-HRMS/MS and label free quantification. The same LC-HRMS setup used for metabolomics experiments was also used for the proteomics analyses. The chromatographic separation was achieved using a reversed phase C_18_ column (Aeris peptide C_18_, 150 × 2.1 mm, 2.6 μm, Phenomenex, Torrance, CA, USA) kept at 30 °C. Mobile phases were water (A) and ACN (B), both containing 0.1% formic acid (*v*/*v*). The chromatographic gradient was: 0–1 min, 2.5% B; 1–20 min, 2.5–30% B; 20–24 min, 30–50% B; 24–26 min, 50–95% B; 26–30 min, 95% B; 30–30.5 min, 95–2.5% B; 30.5–35 min, 2.5% B. Injection volume was 5 μL and flow rate was 0.2 mL min^−1^.

Data were acquired in full-scan data-dependent acquisition mode. Full-scan spectra were acquired at a resolving power of 70,000 FWHM at 200 *m*/*z*. Mass range was between 300 and 2000 *m*/*z*, automatic gain control target was set to 3×10^6^ ions, and inject time was set to 250 ms. Data-dependent fragmentation spectra of the four most intense ions were acquired at a resolution of 17,500, using an isolation window of 1.6 *m*/*z*, automatic gain control target of 2×10^5^, inject time of 120 ms, normalized collision energy of 27, and dynamic exclusion of 30 sec.

Source parameters were as follows: sheath gas flow rate was 25 arbitrary units and auxiliary gas flow rate was 10 arbitrary units. Capillary temperature and heater temperature were set to 325 °C. The capillary voltage was 3.0 kV in positive polarity electrospray, and the S-lens RF level was 55 V.

LC-HRMS/MS data were processed using Proteome Discoverer software (version 2.1) and analyzed with SEQUEST. Protein identification settings were as follows: enzyme specificity set to trypsin with up to 2 allowed missed cleavages, peptide mass tolerance set to 10 ppm, and fragment mass tolerance set to 0.05 Da. Oxidation of methionine and acetylation of *N*-terminus were set as variable modifications, while carbamidomethylation of cysteines was set as fixed modification.

Searches were done using the *Rattus norvegicus* database (UP000002494), and Percolator was used to assess the confidence of peptide and protein identification. Peptides were considered identified with a maximum allowed false discovery rate of 5%, and proteins were considered identified when at least two independent peptides with a confidence level of 95% were present. According to the principle of maximum parsimony, proteins were grouped into protein families. Label-free quantification was achieved using the precursor area detector function, and only unique peptides were used for relative quantification. Protein area values were exported into an Excel spreadsheet for further analysis.

### 2.8. Bioinformatic Analysis

Cluster analysis of serum samples was performed using MeV software (version 4.9.0, SourceForge, San Diego, CA, USA) by construction of the heat map with hierarchical clustering based on the Euclidean distance with average linkage using normalized area values of the altered proteins.

Gene Ontology (GO) enrichment analysis on differentially expressed proteins was performed with EnrichR webtool (Ma’ayan Lab, Icahn School of Medicine at Mount Sinai, New York, NY, USA [38]) using default parameters.

### 2.9. Statistical Analysis

Metabolomics data were analyzed by principal component analysis (PCA) using SIMCA-P software (version 13.0, Umetrics, Umeå, Sweden) to visualize sample grouping and distribution. Normalized intensity of metabolites was mean-centered and variance was set to 1 before performing PCA.

Statistical analysis of the metabolomics and proteomics data was performed using GraphPad Prism version 7.0.0 (San Diego, California, USA) and Microsoft Excel (Microsoft Corporation, Redmond, WA, USA) software; one-way ANOVA was followed by Fisher’s post hoc comparison. Metabolites and proteins showing a *p*-value < 0.05 were considered statistically significant.

## 3. Results

### 3.1. Effects of TM and Captopril Supplementation

We compared the effects of three different dietary conditions on SHRs and the control WKY rats: (i) rats received a standard diet (SD), (ii) rats received a standard diet supplemented with proteins from TM, (iii) rats received a standard diet supplemented with captopril (C). The time-course of systolic blood pressure changes during the 4 weeks of treatment were evaluated in order to assess the effectiveness of the two treatments.

**Figure 1:**
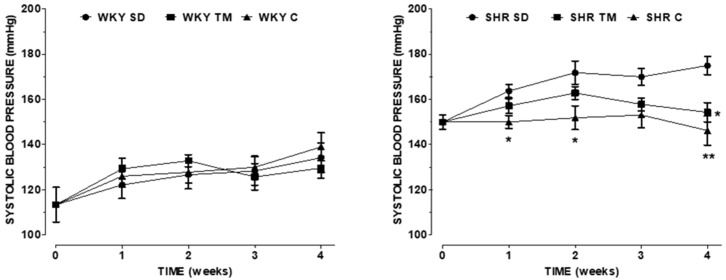
Systolic blood pressure measured during the experimental trial in the six groups under investigation. Left panel: WKY rats; right panel: SHRs. * *p* < 0.05, ** *p* < 0.01, vs. SD at the same time (two-way ANOVA followed by Bonferroni post-test). SD: standard diet; C: captopril; TM: *Tenebrio molitor*.

No differences in blood pressure were observed before the beginning of the treatments in SHRs of the assigned groups. During the experimental trial, the systolic blood pressure increased in SHR-SD, but supplementation with TM proteins (SHR-TM) or captopril treatment (SHR-C) prevented this effect (Figure 1).

However, while captopril immediately lowered the systolic blood pressure, thus supporting its well-known antihypertensive effects, the supplementation with TM proteins was effective only at the end of the 4-week experimental trial. Interestingly, no significant difference in systolic blood pressure between SHR-TM and SHR-C was observed at the third and fourth week of treatment, thus indicating a long-term efficacy of a TM-enriched diet. As a control, there was no effect on systolic blood pressure in the normotensive WKY rats regardless of the administered diets.

To assess the mechanisms occurring at the metabolite or protein level, we first compared SHR-SD and WKY-SD rats.

### 3.2. Metabolomics Profiling of Serum by LC-HRMS

**Figure 2:**
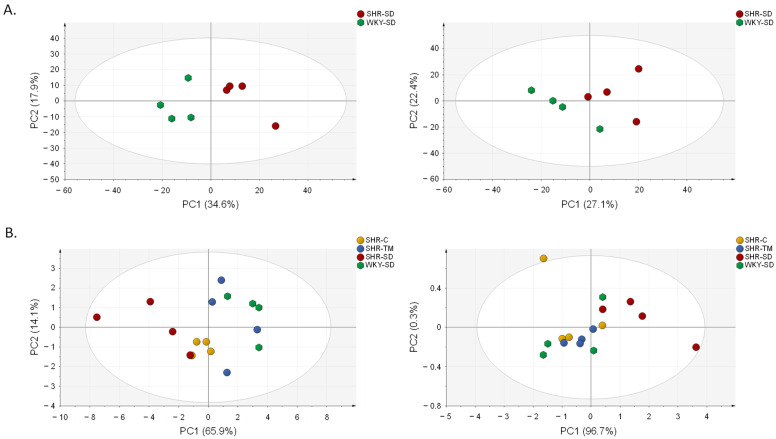
(**A**) Unsupervised PCA performed on metabolomics data from SHR-SD and WKY-SD rats acquired in negative (left) and positive ionization mode (right). The groups are clearly separate on the basis of their global metabolomics profile. (**B**) Unsupervised PCA performed on quantitative data from 15 metabolite targets of intervention by supplementation with protein extracts from TM, 13 of which were acquired in negative (left) and 2 in positive ionization mode (right). Animals of the SHR-SD group are clustered separately from SHR-C and SHR-TM along the first component, indicating that these two groups have a metabolomics pattern closer to that of WKY-SD than to that of SHR-SD.

To evaluate potential differences in metabolite patterns between WKY-SD and SHR-SD rats, we preliminarily applied a metabolomics approach by means of LC–HRMS using both positive and negative ionization polarity. The WKY-SD and SHR-SD metabolite datasets consisted of 934 and 750 ions consistently detected for positive and negative ionization modes, respectively. An unsupervised multivariate principal component analysis (PCA) was used to give a general overview of the bidimensional distribution of the WKY-SD and SHR-SD samples and to display sample grouping. The PCA score plot clearly separated the WKY-SD and the SHR-SD groups both in positive and negative ionization polarity, implying that the global metabolic profile is different between the two groups under comparison (Figure 2A).

Metabolites significantly contributing to the discrimination between the WKY-SD and SHR-SD groups were selected according to results from one-way ANOVA and to their relative serum abundance expressed as the ratio between SHR-SD and WKY-SD rats (SHR/WKY ratio < 0.5 or > 2.0). In total, 38 out of 934 detected signals in positive ionization polarity and 95 out of 750 detected signals in negative ionization polarity were deregulated in the SHR-SD group and thus were potentially correlated to hypertension. We then compared WKY-SD to SHR-SD, SHR-TM, and SHR-C by setting a threshold of statistical significance (i.e., *p* < 0.05) to identify specific molecular targets of TM supplementation among the metabolite markers of hypertension. Fifteen endogenous metabolites were changed significantly toward physiological levels following TM supplementation: four of these biomarkers were down-regulated and eleven were up-regulated in SHR-SD with respect to WKY-SD.

**Figure 3:**
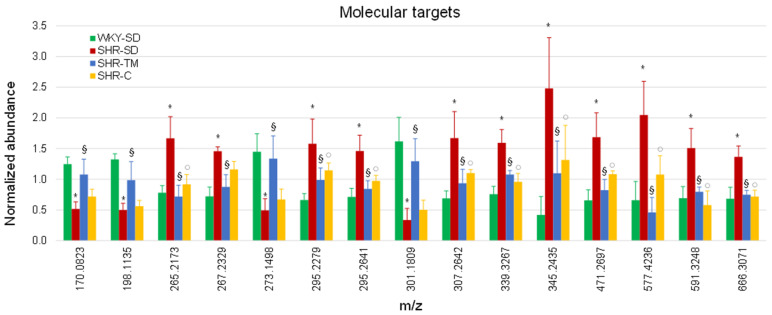
Bar diagram reporting normalized intensity values of metabolite markers of hypertension reversed by supplementation with TM protein extract. * SHR-SD vs. WKY-SD, *p* < 0.05; § SHR-SD vs. SHR-TM, *p* < 0.05; ° SHR-SD vs. SHR-C, *p* < 0.05, one-way ANOVA.

We performed an additional PCA using relative quantification values deriving from these 15 targets of intervention to compare WKY-SD, SHR-SD, SHR-TM, and SHR-C. The PCA score plot showed that SHR-SD was separated from WKY-SD (Figure 2B), while SHR-TM and SHR-C were closer to WKY-SD than to SHR-SD, indicating an almost complete reversion of the 15 molecular targets. The relative abundance of the 15 metabolites is reported in a bar graph showing the WKY-SD, SHR-SD, SHR-TM, and SHR-C groups (Figure 3). Ten of the fifteen metabolites were reversed toward the physiological level by both TM supplementation and captopril administration. On the other hand, no significant differences were observed when WKY-SD rats were compared with WKY rats supplemented with TM protein extracts (WKY-TM) or with WKY rats receiving captopril (WKY-C) (Figure S1).

### 3.3. Annotation of Metabolite Markers

To identify potential targets of action by protein extracts derived from *Tenebrio molitor*, we further characterized the 15 metabolites whose serum levels were altered in SHR-SD and reversed in SHR-TM (Table 1).

These biomarkers were annotated by searching for the measured *m*/*z* value of the precursor ion together with the MS/MS fragmentation spectra in the METLIN, LIPID MAPS, HMDB, and KEGG databases. One of the nine annotated metabolites was identified by comparison of the MS/MS fragmentation spectra, *m*/*z* value of the precursor ion, and chromatographic retention time with authentic reference material (Figure S2). Our results indicate that metabolites of different classes involved in steroid hormone biosynthesis, biosynthesis of unsaturated fatty acids, and amino acid metabolism show marked perturbations in SHR-SD with respect to WKY-SD rats and could contribute to the pathological development of hypertension.

### 3.4. Proteomics Profiling of Serum by LC-HRMS/MS

The proteomics profiling of rat serum was carried out by a shotgun proteomics approach using label-free quantification. Digested proteins from rat serum were separated using RPLC coupled with HRMS/MS. As in the metabolomics analysis, the preliminary analysis of protein expression values compared the WKY-SD and SHR-SD groups to identify the differentially expressed proteins of interest for their potential role in disease progression or as possible pharmacological targets. We set a threshold for the SHR-SD/WKY-SD ratio value as <0.5 or >2 and one-way ANOVA *p*-value < 0.05. In total, 2420 peptides and 219 proteins were identified and relatively quantified (Supplementary Table S1). We found 41 proteins significantly deregulated in SHR-SD with respect to WKY-SD. Among the 41 potential markers of hypertension, 17 proteins were reversed toward the normal levels in SHR-TM after supplementation with TM proteins (*p* < 0.05) (Table 2).

**Figure 4:**
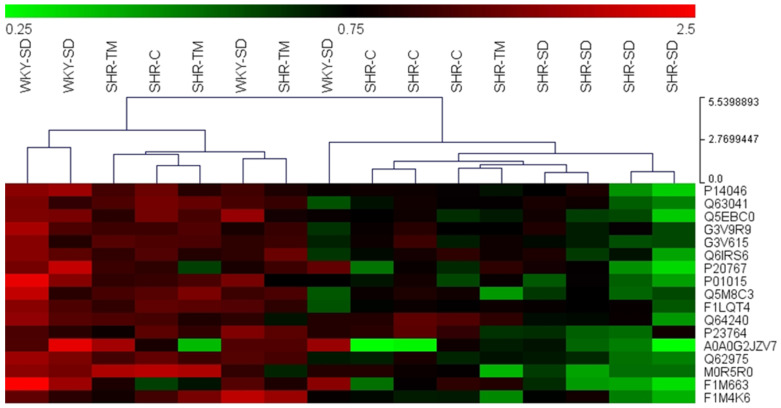
Heat map reporting 17 protein targets of intervention by supplementation with protein extracts from TM. Rows: protein accession number (UniProtKB); columns: animal genotype and experimental groups; color key indicates relative protein amount, red: up-trend; green: down-trend. The protein amount was average normalized.

The normalized relative expression level of the 17 protein markers of intervention were mean-centered and plotted on a heat map for hierarchical clustering, which clearly distinguished SHR-SD from the other groups (i.e., WKY-SD, SHR-TM and SHR-C). This result indicated that both captopril treatment and TM protein extract supplementation restored the protein levels of these markers of hypertension toward the physiological amounts (Figure 4). Indeed, the SHR-SD protein pattern was completely different from those of the other groups, with complete separation of SHR-SD into a distinct cluster on the right of the map.

A Gene Ontology (GO) enrichment analysis (performed using the EnrichR webtool) was carried out to reveal processes in which altered proteins are involved. This analysis showed that biological processes such as “negative regulation of peptidase activity”, “regulation of complement activation”, and “response to hydroperoxide” are downmodulated in SHR animals and restored by TM dietary supplementation (Supplementary Table S2).

## 4. Discussion

It was previously demonstrated that *Tenebrio molitor* represents not only a source of digestible proteins and essential amino acids [28], but it contains molecules endowed with antihypertensive activity [27]. Such hallmarks perfectly overlap with a therapy based on a nutritional approach and support the use of TM as a functional food.

The antihypertensive effect of the TM protein extract can probably be ascribed to the several inhibitory peptides of ACE identified in TM protein extract [39,40] which, by inhibiting ACE activity, interfere with the renin–angiotensin–aldosterone system, one of the most important hormonal mechanisms regulating blood pressure, fluid volume, and sodium–potassium balance [41]. In addition, we have to consider that TM contains GABA among its bioactive nutrients (3.5 mg/100 g of TM, [42]), and that this neurotransmitter decreases blood pressure in both normotensive WKY and SHRs when centrally injected or intraduodenally administered [43,44]. Despite the ability of GABA per se to cross the blood–brain barrier still being debated [45,46], the possibility of a GABA-mediated effect in TM SHR-feeds rats can be reasonably advanced. Considering both the oral intake of GABA (i.e., approximately 0.1 mg/kg, extrapolated by taking into account the daily consumption of TM [27] and the above-reported GABA content) and previous results showing that GABA inhibits blood pressure elevation at a concentration ranging between 0.3 and 1 mg/kg (i.e., higher than the intake of GABA of the present study) [44,47,48], it is unlikely that GABA is responsible for the TM diet-mediated hypotensive effects observed in SHRs.

Nonetheless, in our experiments, the reduction of systolic blood pressure in the SHR animal model after 4 weeks of treatment with a nutritional approach based on dietary supplementation with TM extracts was comparable with that of the known antihypertensive drug, captopril.

Captopril is a widely prescribed drug in clinical medicine to treat hypertension. However, it is not without numerous side effects, from minor paroxysmal cough to hyperkalemia [49]. Hence, the effectiveness of a dietary approach based on the supplementation with edible insects and consumed on a regular basis represents an interesting approach for supporting traditional therapy, allowing the reduction of antihypertensive dosage, and thus mitigating their adverse effects.

In this study we monitored metabolic and proteomic changes in serum from SHRs and age-matched normotensive WKY rats, both subjected either to a TM-supplemented diet or to pharmacological treatment with captopril.

Interestingly, our proteomics data suggest a profound alteration of proteins (8 out of 17) related to the negative inhibition of protease activity. More than 1100 proteases have been found in the human body, involved in several physiological processes ranging from blood coagulation to immunity. Since altered activity of proteases often has pathological consequences, they are strictly regulated by forming a complex with inhibitory proteins, such as the serine protease inhibitor (serpin) family [50].

By comparing the serum of SHR-SD with the WKY-SD counterpart, we found a lower (by at least 60%) amount of angiotensinogen (AGT) in the SHR-SD group. AGT is also known as Serpin A8 [51] whose *N*-terminal cleavage releases angiotensin I, a decapeptide subsequently processed to give the sub-peptides that influence blood pressure [52,53]. However, AGT is not merely an inert substrate, as it was demonstrated to have an active role in the release of angiotensin and in the modulation of blood pressure [54]. Moreover, the reduced level of AGT was restored to the physiological level by both the TM-supplemented diet and by captopril treatment.

AGT is not the only protein of the serpin family whose expression was altered in SHR and restored by a TM-supplemented diet. Indeed, we found perturbed amounts of Serpin A4 and Serpin A10 whose expression is reduced in SHRs and re-established to normal levels with either the pharmacological treatment or the TM-based nutritional approach. Serpin A4, also known as kallistatin, is the endogenous inhibitor of kallikreins, a group of serine proteinases responsible for the cleavage of plasma kininogens to kinins that participates in the regulation of blood pressure. However, kallistatin has many other pleiotropic effects, e.g., by participating in angiogenesis and inflammation processes [55]. The kallistatin level is modified in various pathologies, ranging from liver cirrhosis to cancer and obesity, and it has some protective effects against pathological conditions (e.g., hypertension, cardiovascular damage), as demonstrated by its administration by gene/protein delivery in animal models [55].

Serpin A10, also called protein Z (PZ)-dependent protease inhibitor (ZPI), is a 72 kDa single-chain anticoagulant glycoprotein essential to regulation of the early phase of the coagulation cascade by inhibiting the activity of coagulation factors Xa and XIa, due to the formation of a complex with PZ [56]. Although to our knowledge, there is no direct correlation between ZPI levels and hypertension, it is well-documented that a hypertensive state, as in SHR rats, may promote a prothrombotic state [57], probably by altering the expression levels of proteins involved in the coagulation cascade. Additionally, a link between hypertension and PZ has been reported for hypertensive patients in whom a low level of the cofactor PZ was observed [58].

Alpha-1-macroglobulin (A1m), another inhibitor of proteinases, and the plasma protein fetuin B, a liver-derived cysteine protease inhibitor [59,60], were found to be profoundly altered in SHR rats; their levels were restored by the two different treatments (i.e., captopril and nutritional approach with TM). Although no information is available about the correlation between fetuin B and hypertension, increasing evidence suggests that alteration of its expression is associated with metabolic dysfunction and also cardiovascular disease [61]. Interestingly, Gonzalez-Marrero and colleagues demonstrated a different proteomic profile of SHRs when compared with the normotensive control rats, with a reduced expression in cerebrospinal fluid of fetuin-A, the most important fetuin-B homolog, which inhibits pathological calcifications by forming complexes with calcium and phosphorus [62].

The α1-microglobulin/bikunin precursor protein (AMBP) was also altered in the SHR-SD group. This protein is usually cleaved into two polypeptides: alpha-1 microglobulin is involved in inflammatory processes, while bikunin is a protease inhibitor related to many physiological and pathological processes [63]. In agreement with our results, alpha-1 microglobulin was previously demonstrated to be a potential therapeutic target for the treatment of hypertension in animal models [64].

Our proteomics data suggest that ProS1, another cofactor necessary for the inactivation of factors Va and VIIIa and whose deficiency is linked to thromboembolism [65], was deregulated in SHR animals. ProS1 expression was restored by the nutritional intervention, thus suggesting that an impairment of the coagulation cascade, which represents a secondary effect of hypertension, could be ameliorated by a TM-enriched diet.

Afamin, a member of the albumin gene family [66], was another protein deregulated in SHRs. It is a vitamin E-binding glycoprotein that circulates at relatively high concentrations in the bloodstream and other body fluids, such as cerebrospinal, ovarian follicular and seminal fluids. It is involved in the transport of vitamin E across the blood–brain barrier, and several studies suggest its involvement in neuroprotection and fertility. Our findings are fully in agreement with previous results indicating that afamin is an excellent early marker of metabolic syndrome and other related pathologies such as hypertension [67,68].

Glutathione peroxidase 3 (GPX3) expression was also found to be reduced in serum of the SHR-SD group with respect to its normotensive counterpart. As observed for other deregulated proteins, treatment with either captopril or TM extract allowed for the complete reversion of GPX3 expression to the physiological condition. It should be noted that GPX3 is a selenoenzyme that catalyzes the reduction of hydrogen peroxide or organic hydroperoxides using glutathione and thioredoxin [69] and it is the major scavenger of reactive oxygen species (ROS) in the blood. The under-expression of GPX3 has been associated with vascular diseases and an increased incidence of ischemic stroke [70,71]. Moreover, hypertension is associated with a blood ROS concentration increase and deficient endogenous antioxidant mechanisms that might be either the cause or the effect of the disease [72,73,74]. Further studies are needed to understand the causes of the reduced expression of GPX3 and how the two treatments alleviate the reduction. However, it is well-established that its expression is regulated by several transcriptional factors or by epigenetic mechanisms (e.g., hypermethylation in CpG islets) [75] that may be altered in SHRs and reversed by the two different treatments used in this study.

In accordance with the proteomics data, the metabolomics results showed an altered amount of epoxyoctadecenoic acid, a peroxidation product of linoleic acid associated with cardiovascular effects, which may be produced by increased ROS levels [76,77]. An altered metabolism of linoleic acid and unsaturated fatty acids was also indicated by the significantly increased amount of eicosadienoic, docosanoic, and nonadecenoic acid in the SHR-SD group. These alterations were completely reversed to physiological levels by both captopril and TM extract. These data are consistent with recent reports demonstrating that hypertension is related to an increased number of oxidants leading to lipid peroxidation increase and formation of by-products, which have been used as biomarkers to evaluate hypertensive patients or experimental hypertension [78].

## 5. Conclusions

In summary, our study on the SHR animal model highlighted metabolic and proteomic dysregulation that can be restored by a nutritional approach based on the introduction of *Tenebrio molitor* protein extract into the diet. The results suggest that such chronic treatment may affect the expression or secretion of proteins fundamental for the regulation of blood enzymes, such as proteases, whose activity is strictly regulated to preserve a healthy condition. However, studies on hypertensive patients will be necessary to validate our findings in humans and to thoroughly investigate the beneficial effects of this promising new food.

## Figures and Tables

**Table 1:** List of metabolite targets of *Tenebrio molitor* protein extract intervention. Measured *m*/*z* value, chromatographic retention time, elemental composition, adduct, annotation, class of metabolite, ionization polarity, and relative intensity value (i.e., SHR-SD/SHR-TM ratio) are reported for each detected signal.

*m*/*z*	Retention Time (min)	Elemental Composition	Adduct	Annotation or Identification *	Class	Ionization Polarity	Ratio SHR-SD/SHR-TM
170.0823	4.7	C_8_H_15_NO_4_	[M-H_2_O-H]^−^	hydroxyhexanoylglycine	acyl aminoacids	Negative	0.48
198.1135	7.2	C_10_H_19_NO_4_	[M-H_2_O-H]^−^	propionylcarnitine	short chain acylcarnitine	Negative	0.51
265.2173	13.6		-	-	-	Negative	2.32
267.2329	14.3		-	-	-	Negative	1.66
273.1498	8.7		-	-	-	Negative	0.37
295.2279	11.1	C_18_H_32_O_3_	[M-H]^−^	epoxyoctadecenoic acid	peroxidation product of linoleic acid	Negative	1.60
295.2641	15.2	C_19_H_36_O_2_	[M-H]^−^	nonadecenoic acid	long chain fatty acids	Negative	1.73
301.1809	10.2	C_19_H_26_O_3_	[M-H]^−^	19-hydroxyandrost-4-ene-3,17-dione	steroid	Negative	0.26
307.2642	14.9	C_20_H_36_O_2_	[M-H]^−^	eicosadienoic acid	unsaturated fatty acids	Negative	1.79
339.3267	16.9	C_22_H_44_O_2_	[M-H]^-^	docosanoic acid *	unsaturated fatty acids	Negative	1.48
345.2435	12.1		-	-	-	Negative	2.25
471.2697	14.9	C_11_H_19_NO_3_	[2M+HCOO]^−^	nonenoylglycine	acyl aminoacids	Negative	2.05
577.4236	13.4	C_31_H_63_O_7_P	[M-H]^−^	1-hexadecyl-2-dodecanoyl-glycero-3-phosphate	glycerophospholipids	Negative	4.45
591.3248	11.6	C_27_H_39_N_7_O_7_	[M+NH_4_]^+^	-	peptides	Positive	1.89
666.3071	11.6	C_28_H_41_N_11_O_7_	[M+Na]^+^	-	peptides	Positive	1.83

* identified by comparison with reference material.

**Table 2:** List of 17 identified and relatively quantified proteins reversed by *Tenebrio molitor* supplementation in SHR-TM rats. The table reports the UniProtKB accession number, gene name, description, sequence coverage (%), number of unique peptides, and relative expression value (i.e., SHR-SD/SHR-TM ratio).

UniProtKB Accession	Description	Gene Name	Sequence Coverage (%)	Unique Peptides	Ratio SHR-SD/SHR-TM
P14046	Alpha-1-inhibitor 3	A1i3	64.0	31	0.33
Q5EBC0	Inter alpha-trypsin inhibitor, heavy chain 4	Itih4	73.3	107	0.32
Q6IRS6	Fetub protein	Fetub	62.7	47	0.43
P20767	Ig lambda-2 chain C region	ENSRNOG00000050000	64.5	45	0.31
P01015	Angiotensinogen	Agt	48.6	36	0.28
F1LQT4	Carboxypeptidase N subunit 2	Cpn2	64.6	20	0.51
Q64240	Protein AMBP	Ambp	89.4	9	0.46
A0A0G2JZV7	Ig-like domain-containing protein	ENSRNOG00000047464	42.1	15	0.42
Q62975	Protein Z-dependent protease inhibitor	Serpina10	39.7	12	0.44
M0R5R0	Protein S (Alpha), isoform CRA_b	Pros1	32.2	13	0.36
F1M663	Ig-like domain-containing protein	ENSRNOG00000048017	24.4	8	0.28
F1M4K6	Leucine-rich repeat-containing protein 7	Lrrc7	36.3	8	0.36
Q63041	Alpha-1-macroglobulin	A1m	52.3	5	0.49
G3V9R9	Afamin	Afm	13.5	4	0.51
G3V615	Complement factor B	Cfb	13.9	5	0.51
Q5M8C3	Serine (Or cysteine) proteinase inhibitor, clade A (Alpha-1 anti-proteinase, antitrypsin), member 4	Serpina4	6.5	5	0.42
P23764	Glutathione peroxidase 3	Gpx3	33.9	3	0.45

## Data Availability

The mass spectrometry proteomics data have been deposited with the ProteomeXchange Consortium via the PRIDE partner repository with identifier PXD034456.

## Supplementary Materials

The following supporting information can be downloaded at: https://www.mdpi.com/article/10.3390/nu14163288/s1, Figure S1. Effects of different diets on WKY rats; Figure S2. Identification of docosanoic acid; Table S1. List of identified proteins; Table S2. Gene Ontology (GO) enrichment analysis.
