# Supplementary material for: Serum Metabolomics and Proteomics to Study the Antihypertensive Effect of Protein Extracts from Tenebrio molitor
Source: Nutrients. 2022 Aug 11;14(16):3288. doi: 10.3390/nu14163288 (PMC9413627; doi:10.3390/nu14163288)
Supplement: Supplementary file 1 [file nutrients-14-03288-s001.zip › Supplementary_Material.pdf]

Supplementary Material to "Serum metabolomics and proteomics to study the antihyper-tensive effect of *Tenebrio molitor* extracts".

Supplementary Figure S1. Effects of different diets on WKY rats. Bar diagram reporting normalized intensity values of metabolite markers of hypertension reversed in SHR after supplementation with TM protein extract. No significant difference were observed by applying one-way ANOVA.

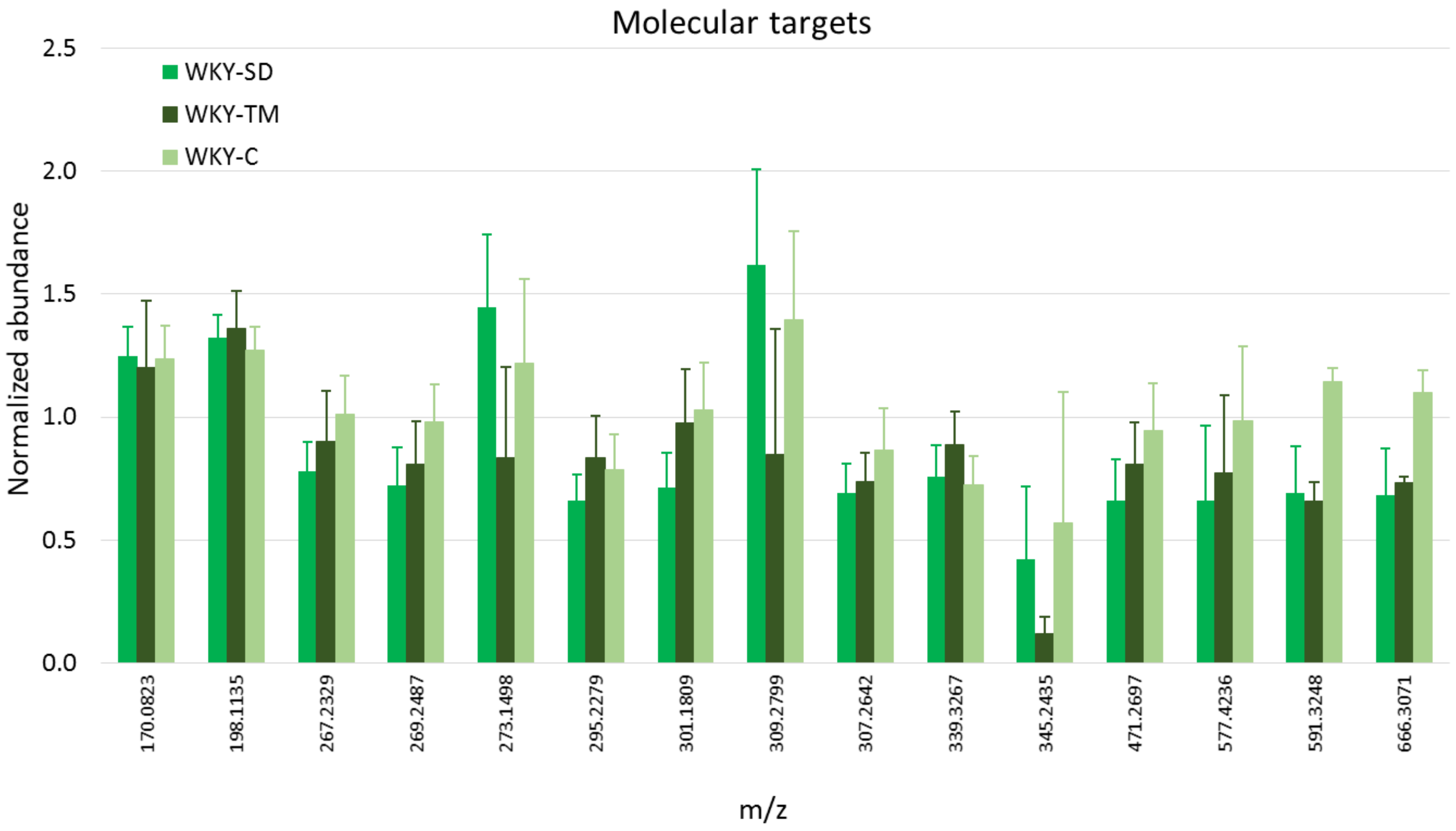

Supplementary Figure S2. Identification of docosanoic acid. LC-HRMS/MS analysis of metabolite with *m/z* signal of 339.3267. Extracted HRMS ion chromatogram (left panel), full scan spectrum of precursor ion (center panel) and HRMS/MS spectrum of *m/z* 339.3267 reporting product ions (right panel). The metabolite was confirmed as docosanoic acid by comparison of chromatographic retention time, isotopic pattern and product ions to that of a reference standard.

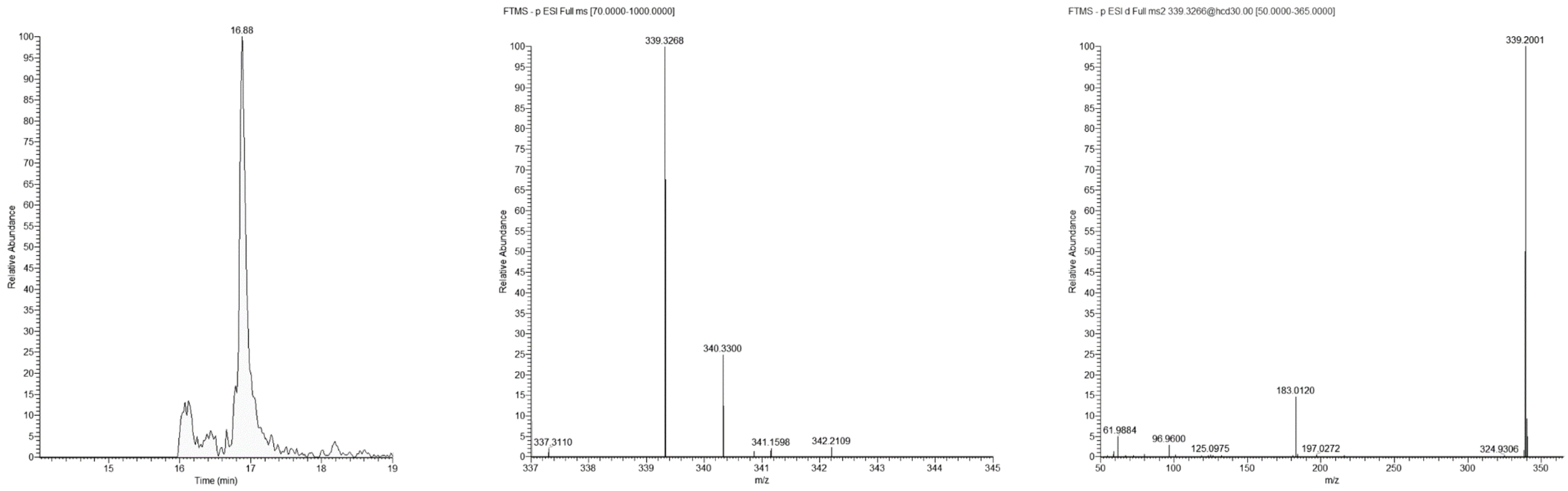

Supplementary Table S2. **Gene Ontology (GO) enrichment analysis.** The GO enrichment analysis was performed using the EnrichR webtool to reveal processes in which altered proteins in SHR-SD with respect to WKY-SD rats are involved. In the table, it is reported the *p*-value, the adjusted *p*-value and the proteins involved for each term. Only statistically significant terms were listed.

Term	*p* -value	Adjusted *p* -value	Odds Ratio	Combined Score	Gene name
negative regulation of peptidase activity (GO:0010466)	1.19E-07	8.44E-06	1.23E+02	1958.257685	SERPINA10;FETUB;AGT;SERPINA4
negative regulation of endopeptidase activity (GO:0010951)	1.19E-07	8.44E-06	1.23E+02	1958.257685	SERPINA10;FETUB;AGT;SERPINA4
regulation of endopeptidase activity (GO:0052548)	1.93E-07	9.14E-06	1.08E+02	1671.228941	SERPINA10;FETUB;AGT;SERPINA4
regulation of complement activation (GO:0030449)	6.55E-06	1.96E-04	1.06E+02	1265.872335	PROS1;CPN2;CFB
regulation of immune effector process (GO:0002697)	7.82E-06	1.96E-04	9.97E+01	1172.101688	PROS1;CPN2;CFB
regulation of humoral immune response (GO:0002920)	8.27E-06	1.96E-04	9.77E+01	1143.520334	PROS1;CPN2;CFB
platelet degranulation (GO:0002576)	1.03E-04	2.08E-03	4.07E+01	373.8244999	ITIH4;PROS1;SERPINA4
regulated exocytosis (GO:0045055)	3.01E-04	5.35E-03	2.80E+01	226.8315097	ITIH4;PROS1;SERPINA4
positive regulation of gap junction assembly (GO:1903598)	4.49E-03	4.96E-02	2.85E+02	1542.867593	AGT
regulation of extracellular matrix assembly (GO:1901201)	5.24E-03	4.96E-02	2.38E+02	1249.077685	AGT
positive regulation of activation of Janus kinase activity (GO:0010536)	5.24E-03	4.96E-02	2.38E+02	1249.077685	AGT
regulation of NAD(P)H oxidase activity (GO:0033860)	5.24E-03	4.96E-02	2.38E+02	1249.077685	AGT
regulation of neurotrophin TRK receptor signaling pathway (GO:0051386)	5.24E-03	4.96E-02	2.38E+02	1249.077685	AGT
regulation of systemic arterial blood pressure by renin-angiotensin (GO:0003081)	5.24E-03	4.96E-02	2.38E+02	1249.077685	AGT
complement activation, alternative pathway (GO:0006957)	5.24E-03	4.96E-02	2.38E+02	1249.077685	CFB
response to hydroperoxide (GO:0033194)	5.99E-03	5.00E-02	2.04E+02	1043.434298	GPX3
regulation of activation of Janus kinase activity (GO:0010533)	5.99E-03	5.00E-02	2.04E+02	1043.434298	AGT
